# Recent advances in near-infrared I/II persistent luminescent nanoparticles for biosensing and bioimaging in cancer analysis

**DOI:** 10.1007/s00216-024-05267-z

**Published:** 2024-04-09

**Authors:** Ming-Hsien Chan, Yu-Chan Chang

**Affiliations:** Department of Biomedical Imaging and Radiological Sciences, National Yang Ming Chiao Tung University, 112304 Taipei, Taiwan

**Keywords:** Near-infrared I/II, Persistent luminescent nanoparticles, Biosensors, Bioimaging

## Abstract

Photoluminescent materials (PLNs) are photoluminescent materials that can absorb external excitation light, store it, and slowly release it in the form of light in the dark to achieve long-term luminescence. Developing near-infrared (NIR) PLNs is critical to improving long-afterglow luminescent materials. Because they excite *in vitro*, NIR-PLNs have the potential to avoid interference from *in vivo* autofluorescence in biomedical applications. These materials are promising for biosensing and bioimaging applications by exploiting the near-infrared biological window. First, we discuss the biomedical applications of PLNs in the first near-infrared window (NIR-I, 700–900 nm), which have been widely developed and specifically introduce biosensors and imaging reagents. However, the light in this area still suffers from significant light scattering and tissue autofluorescence, which will affect the imaging quality. Over time, fluorescence imaging technology in the second near-infrared window (NIR-II, 1000–1700 nm) has also begun to develop rapidly. NIR-II fluorescence imaging has the advantages of low light scattering loss, high tissue penetration depth, high imaging resolution, and high signal-to-noise ratio, and it shows broad application prospects in biological analysis and medical diagnosis. This critical review collected and sorted articles from the past 5 years and introduced their respective fluorescence imaging technologies and backgrounds based on the definitions of NIR-I and NIR-II. We also analyzed the current advantages and dilemmas that remain to be solved. Herein, we also suggested specific approaches NIR-PLNs can use to improve the quality and be more applicable in cancer research.

**Graphical abstract:**

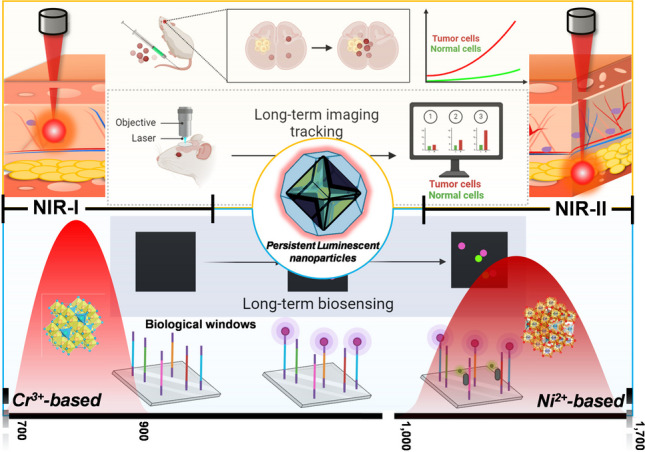

## Introduction

In recent years, inorganic nanomaterials, such as quantum dots, upconversion nanoparticles, and persistent luminescent nanoparticles (PLNs), have been widely used in biomedical imaging and therapy. Traditional high-temperature solid-phase synthesis methods for persistent luminescent phosphors are not suitable for biomedical applications due to poor biocompatibility and large particle sizes [1]. Consequently, new synthesis methods for PLNs, including sol-gel processes, solvothermal methods, and template methods, have been proposed [2]. Compared to conventional materials, nanomaterials exhibit distinct physicochemical properties, characterized by larger surface areas and tunable microstructures. These features provide nanomaterials with greater diversity and applicability. Coupled with medical technologies, they contribute to more precise diagnostics and rapid detection and treatment of diseases. The porous structure, hollow configuration, large surface area, and surface charge of nanoparticles show potential for drug delivery, overcoming limitations associated with small-molecule drugs [3]. This approach prevents premature degradation of drugs and enables controlled drug loading and release. Additionally, nanomaterials can release drugs in response to temperature, light, pH changes, ultrasound, and other stimuli, achieving targeted therapeutic effects through surface modifications with specific molecules or functional groups [4].

In the medical field, there has been a concerted effort to develop methods for faster disease detection; the aim is to develop tools that are rapid, cost-effective, comfortable, highly sensitive, and portable for real-time tracking and monitoring. Commonly used diagnostic tools include X-ray imaging and magnetic resonance imaging (MRI), which provide rapid visualization and functional insights into biological structures and responses. Leveraging the unique optical properties of nanoparticles enables real-time imaging and tracking within living organisms. Nanoparticles (NPs) can be classified into upconversion NPs, downconversion NPs, and PLNs; PLNs exhibit stable luminescence for extended periods, avoiding autofluorescence and reducing noise interference. PLNs that emit in the infrared spectrum are particularly useful in biomedical applications, such as cellular tracking and phototherapy [5]. To enhance the precision in diagnosis and treatment, multiple imaging modalities are a current focus of research. The contribution of nanomaterials to medicine is evident, and the application of this technology to tumor imaging and biosensing is desirable [6]. By harnessing the multifunctionality and transport capabilities of composite nanoparticles, the goal is to increase the efficiency of analysis and achieve multifunctional detection effects [7]. The key distinctions between PLNs and other luminescent nanomaterials are their ability to emit light continuously for an extended period, respond to various light sources with multiple excitation bands, and exhibit repetitive luminescence. When PLNs are excited by ultraviolet light, long-term NIR emission can prevent interference from endogenous fluorescence in biological systems. These probes demonstrate excellent signal-to-noise ratios and deep tissue penetration in *in vitro*, *in vivo*, and *in situ* imaging [8]. Consequently, NIR-PLNs can be utilized for tracking tumor locations for diagnostic and therapeutic purposes [9].

**Fig. 1:**
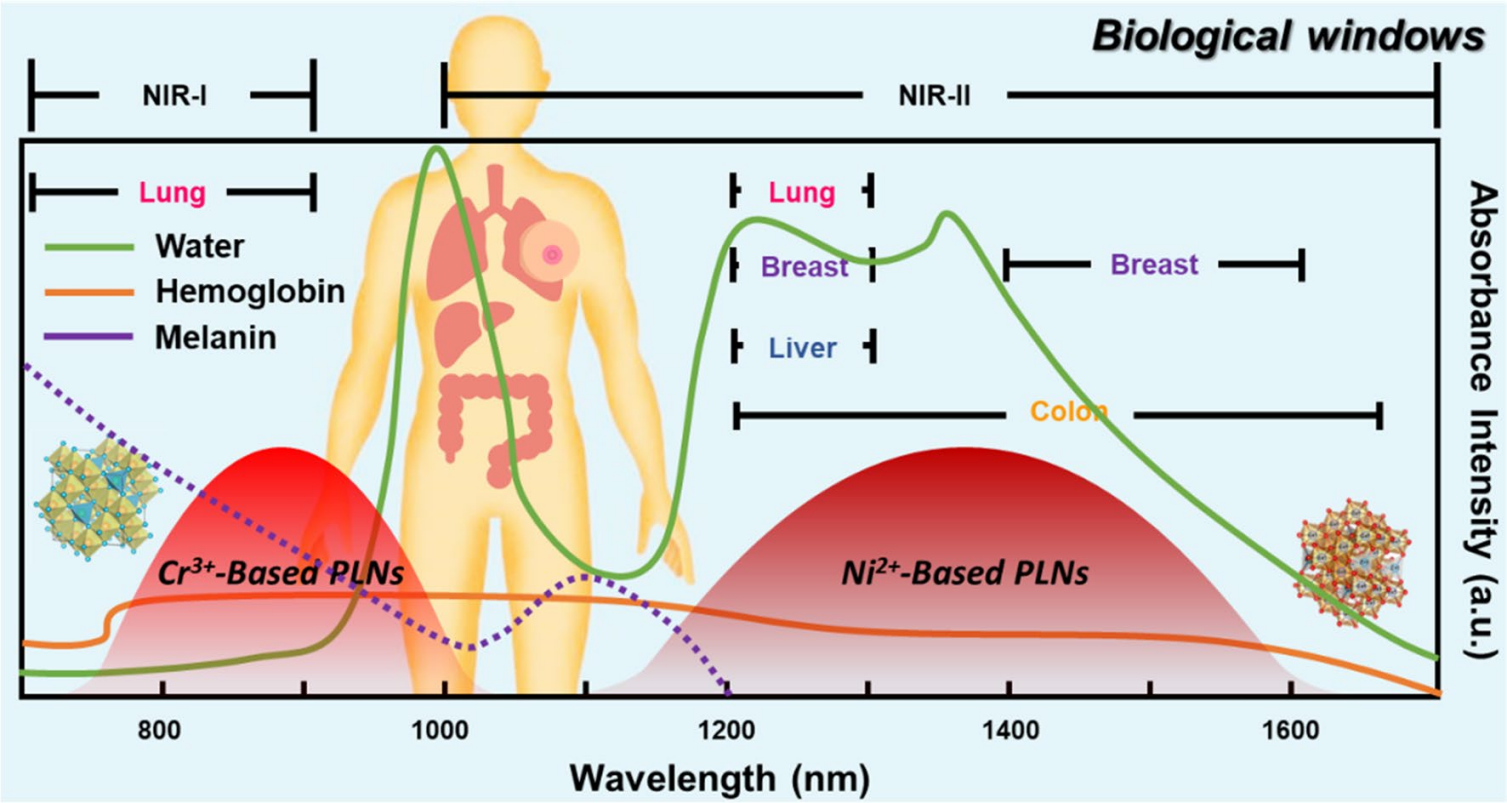
The range of the optical window in biological tissues illustrates a specific NIR window

The primary concern in optical imaging of biological tissues lies in the selection of the light source [10]. Within the wavelength range from the visible to the NIR region, scattering is the predominant interaction between light and tissues, causing rapid diffusion of light during propagation [11]. Increased scattering results in a greater probability of photon absorption within the tissue. In practice, the scattering effect minimally varies with wavelength; thus, the range of the optical window for biological tissues is constrained by the absorption of specific substances, primarily water, hemoglobin in the blood, blood glucose, skin pigments, myoglobin, and cellular pigments. Since hemoglobin exhibits high absorption bands at wavelengths less than 600 nm, the lower limit (short wavelength) of the biological tissue window is determined by blood absorption [12]. Conversely, the upper limit (long wavelength) is determined by water absorption, particularly when the wavelength exceeds 1150 nm. In comparison to visible light, NIR light is minimally absorbed, is scattered less, and has higher transmittance in biological tissues [13]. This reduces the likelihood of tissue burns. Selecting an appropriate light source within the wavelength range of the optical window enhances imaging or therapeutic efficiency, increases penetration depth, and reduces light-induced tissue damage in applications such as optical imaging and photothermal therapy [14]. The NIR window included three biological therapeutic windows: the first NIR window (650–950 nm, NIR-I), the second NIR window (1000–1350 nm, NIR-II), and the third NIR window (1550–1870 nm, NIR-III), as illustrated in Fig. 1. Based on the absorption and reflection characteristics of organs, the NIR-I window is suitable for biological applications related to the lungs and brain. The NIR-II window is suitable for analysis of the chest, colon, rectum, lungs, and liver. The NIR-III window is suitable for biological applications related to the chest, colon, rectum, and thyroid.

For instance, based on the treatment of the brain, we focused on the absorption and reflection characteristics of the skin tissue overlying the brain, skull, and cerebral cortex [15]. The investigation revealed that no significant difference was observed in tissue penetration among the different NIR windows for the extracranial tissues [16]. Upon comprehensive evaluation, NIR-I and NIR-II were selected as the basis for selecting fluorescent labeling materials. This review provides an in-depth exploration of various synthesis techniques for preparing PLN molecular probes and their use as targeted probes for sensing, detection, and *in vivo* imaging after surface modification. This study delves into the application of PLN luminescent materials doped with Mn^2+^ and Cr^3+^ transition metal ions, which exhibit strong persistent NIR luminescence, in *in vivo* imaging [17]. Furthermore, the paper discusses the use of these materials as imaging probes for acute and chronic toxicity in different biological systems. In summary, this review provides insight into the challenges and future research directions in the field of biological imaging applications [18]. Another notable application value of NIR-PLNs is based on functionalized nanomaterials; these materials provide a promising technological platform for long-term real-time monitoring of physiological processes in vivo and for diagnosing diseases [19]. This review also provides an in-depth discussion on the different synthesis technologies of long NIR-PLN molecular probes and their surface modifications as targeted probes for sensing detection and *in vivo* imaging in recent years; this especially applies to NIR-I and NIR-II in this specific optical window [20]. Finally, the challenges and future research directions still faced by bioimaging and molecular sensing applications are discussed [21].

## Persistent luminescent nanoparticles and their luminescent mechanism

Afterglow materials have been among the earliest materials studied and applied, with many natural minerals inherently possessing long afterglow luminescent characteristics. These materials have been utilized in the production of various items, such as “luminous cups” and “night pearls.” The earliest documented use may be from the Song Dynasty in China during the reign of Emperor Taizong (976–997 AD), where “long afterglow pigments” were recorded in the creation of a “bull painting.” The bull in the painting could still be viewed at night, which was attributed to the use of luminescent pigments made from oysters. The earliest Western record of these luminescent materials dates back to 1603 when an Italian shoemaker, during the alchemical process of burning local minerals, obtained a substance emitting red light in the dark. Upon analysis, the mineral was found to contain barium sulfate, and after reduction roasting, some of the mineral was transformed into barium sulfide, a long afterglow material. Subsequently, in 1764, an Englishman produced a blue-white luminescent material by burning a mixture of oyster shells and sulfur, namely, calcium sulfide, another long afterglow luminescent material. Compared with conventional materials, nanomaterials exhibit distinctive physicochemical properties, and when combined with medical technologies, their use enhances the precision of diagnostic methods. Moreover, these methods contribute to the rapid detection and subsequent treatment of diseases. By utilizing the unique optical properties of nanoparticles, imaging within biological organisms becomes feasible. Nanoparticles (NPs) can be categorized into upconversion NPs, downconversion NPs, and PLNs based on their different optical characteristics. Among these, PLNs, through their base materials and doping elements, provide a rich electron orbital structure, enabling the temporary storage of stimulated electrons. Consequently, these materials exhibit stable luminescence over an extended period. PLNs emitting in the infrared spectrum are particularly useful in biomedical applications due to the superior ability of these materials to penetrate infrared light, which is commonly utilized for cellular tracking and phototherapy. However, afterglow materials have relatively stable luminescence at the microscale level and not the nanoscale level [22]. Therefore, the preparation of stable and long-lasting luminescent nanoscale afterglow materials, the optimized PLNs, has become an important issue. In this chapter, we will briefly describe the methods used to prepare PLNs and the types of crystal structures currently studied.

### Structures of PLN materials

#### Aluminate-based materials

Matsuzawa et al*.* synthesized Dy-doped SrAl_2_O_4_:Eu in 1993; this material has a remarkably long afterglow decay time of up to 2000 min [23]. Subsequently, a series of rare earth activated aluminate long afterglow materials have been developed; these afterglow materials include the blue CaAl_2_O_4_:Eu,Nd and the blue-green Sr_4_Al_14_O_25_:Eu,Dy. The long afterglow materials and their corresponding performance parameters are summarized in Table 1. In aluminate-based long afterglow materials, the activator is primarily Eu, and the afterglow emission colors are concentrated in the blue-green wavelength range [24]. Despite the relatively poor water resistance of aluminates, aluminate-based long afterglow materials, including SrAl_2_O4:Eu,Dy and Sr_4_Al_14_O_25_:Eu,Dy, have gained significant commercial value, making them the primary focus of research and application in the field.

**Table 1:** Biomedical imaging applications of PLN materials

Biomedical imaging	PLNs material	Modifier	Maximum excitation /emission Ex/Em (nm)	Advantages	Disadvantages	Ref.
Near-infrared-I (650–950 nm) (from 2018 to 2023)	Zn_3_Ga_2_Ge_2_O_10_:Cr^3+^,Eu^3+^ (ZGGO:Cr^3+^,Eu^3+^)	NH_2_	254/697 nm	Excellent imaging capacity for cells *in vitro*	Without *in vivo* imaging	[24]
	Zn_1.1_Ga_1.8_Ge_0.1_O_4_:Cr^3+^ (ZGG)	PEG	254/696 nm	Macrophage and inflammation imaging	Only for RAW 264.7 cell line	[49]
	ZnGa_2_O_4_:Cr^3+^ (ZGC)	PEG	254/700 nm	Solve the problem of the low re-excitation efficiency	Lack of specific cell labeling capabilities	[50]
	PLNPs@ZIF-8	ZIF-8	254/696 nm	A promising theranostic platform	Lack of specific cell labeling capabilities	[51]
	Zn_1+*x*_Ga_2–2*x*_Sn_*x*_O_4_:Cr^3^	n/a	625/696 nm	Higher storage capacity of charge trapping	Without *in vivo* imaging	[22]
	ZnGa_2_O_4_:Cr^3+^, (CZGO)-ACP	ACP	254/695 nm	Bioimaging and biosensing dual functions	Lack of more in-depth biological experimental testing	[17]
	MgGeO_3_:Mn^2+^,Yb^3+^	n/a	295/680 nm	Developing optical data storage technology	Lack of more in-depth biological experimental testing	[18]
	Gd_2.99_Ce_0.01_Al_1.995_Cr_0.005_Ga_3_O_12_ (GAGG:CeCr)	FA	Multiple ex./720 nm	High contrast bioimaging	Without *in vivo* imaging	[52]
	SiO_2_@Zn_1.05_Ga_1.9_O_4_:Cr	n/a	254/696 nm	Tri-mode for clinical cancer therapy	The duration of persistent luminescent is shorter	[53]
	LiGa_5_O_8_:Cr^3+^	Lip	285/718 nm	Dendritic cell tracking	Larger particle size	[54]
	PLNPs-PAMAM	AS1411	254/700 nm	A dendrimer and aptamer grafted particles	Complex surface modification	[55]
	ZnGa_2_O_4_:Cr^3+^/Sn^4+^@MSNs	PEG	254/695 nm	Optimized particle size is suitable for bioimaging	Lack of specific cell labeling capabilities	[56]
	AP-NPs@SiO_2_	PAA	Multiple ex./em.	Diverse excitation and emission light	Without *in vivo* imaging	[19]
	ZGGO: Cr_0.02_,Yx	SiO_2_	296/697 nm	Y^3+^ doping improves persistent intensity	Without *in vivo* imaging	[36]
	LZGG: *m*Cr^3+^,*n*Ti^4+^	n/a	254/700 nm	Systematic investigations of the energy traps	Lack of more in-depth biological experimental testing	[57]
	Zn_1.33_Ga_1.335_Cr_0.005_Sn_0.33_O_4_ (ZGSO:0.5%Cr^3+^)	n/a	450/700 nm	Impact on the morphological feature	Without *in vivo* imaging	[30]
	ZnGa_2_O_4_:Cr^3+^ with Li^+^/Ga^3+^	n/a	Multiple ex./em.	Diverse excitation and emission light	Lack of more in-depth biological experimental testing	[26]
	Zn_2_Ga_3.98-4*x*/3_Ge_*x*_O_8_:Cr_0.02_ (ZGGO:Cr)	n/a	254/700 nm	Bioimaging and biosensing dual functions	Lack of more in-depth biological experimental testing	[58]
Near-infrared-II (1000–1700 nm)	LN-PLN	n/a	X-ray/multiple em.	NIR-II persistent luminescent bioimaging	X-ray-activated	[59]
	ZnGa_2_O_4_:Cr^3+^,Ni^2+^	PEG	440/1285 nm		The duration of persistent luminescent is shorter	[60]
	Y_3_(Al/Ga)_5_O_12_:Ce^3+^,Cr^3+^,Nd^3+^	n/a	410/1063 nm		Without *in vivo* imaging	[61]
	MgGe_0.8_Ga_0.2_O_3_:Yb^3+^	n/a	X-ray/950–1100 nm		Without *in vivo* imaging	[62]
	mSiO_2_@Gd_3_Ga_5_O_12_:Cr^3+^,Nd^3+^	n/a	808/1067 nm		Lack of specific cell labeling capabilities	[63]

^*^n/a means “Not applicable”

#### Silicate-based materials

Silicate-based long afterglow materials utilizing silicate as a substrate have garnered increasing attention in recent years due to the excellent chemical and thermal stability of silicates, along with the cost-effective and readily available raw material SiO_2_. Since the development of the silicate long afterglow material Zn_2_SiO_4_:Mn [25], as in Japan in 1975, with an afterglow time of 30 min, various silicate long afterglow materials have been developed. Examples include Sr_2_MgSi_2_O_7_:Eu,Dy, Ca_2_MgSi_2_O_7_:Eu,Dy, and MgSiO_3_:Mn,Eu,Dy, with the materials and performance parameters presented in Table 1. The primary activator in silicate-based long afterglow materials is Eu^2+^, with emission colors predominantly in the blue-green range. Although red-emitting silicate long afterglow materials have been reported, those with superior afterglow performance include Sr_2_MgSi_2_O_7_ and Ca_2_MgSi_2_O_7_ co-doped with Eu and Dy; these exhibit afterglow durations exceeding 20 h. Additionally, red afterglow phenomena have been observed in MgSiO_3_ co-doped with Mn, Eu, and Dy. Silicate-based long afterglow materials provide superior water resistance compared to aluminate-based materials, although their overall performance may be inferior.

#### Others

In 2017, Jing Wang and his team successfully synthesized PLNs labeled as ZnGa_2_O_4_@MSN@Gd_2_O_3_. Through a high-temperature calcination process, Zn^2+^, Ga^3+^, Cr^3+^, and Sn^4+^ were incorporated into mesoporous silica nanoparticles (MSNs) [26]. Subsequently, Gd^3+^ was encapsulated in the outer layer of ZnGa_2_O_4_@MSN using another high-temperature calcination step, resulting in the formation of PLNs with an outer coating of Gd_2_O_3_ (ZnGa_2_O_4_@MSN@Gd_2_O_3_). This particle system simultaneously exhibited dual functionality for both magnetic resonance imaging (MRI) and NIR imaging [27]. Following surface modification, targeted imaging within biological organisms could be achieved; thus, this technique is a versatile tool for biomedical applications. The distinctive feature of this method is the utilization of mesoporous silica nanoparticles (MSNs) as carriers. MSNs, composed of SiO_2_, exhibit high thermal and hydrothermal stability. At the nanoscale, MSNs possess large pore volumes, enabling the loading of substantial amounts of samples or drugs. ZnGa_2_O_4_ has a high luminescence capability and prolonged afterglow duration, enabling the conversion of visible light to NIR-I light. Therefore, ZnGa_2_O_4_ is suitable for application in biological imaging. ZnGa_2_O_4_ has a cubic spinel structure AB_2_O_4_, representing a chemically and thermally stable wide bandgap semiconductor. In the spinel structure, A is typically a divalent ion (Zn, Mg, Ca, and Sr), while B is a trivalent ion (Al, Ga, and Cr). In ordinary spinels, zinc ions (A) are coordinated tetrahedrally with oxygen atoms, and gallium ions (B) occupy octahedral positions. When transition metal elements are doped, various luminescent signals are observed. Specifically, doping with Cr^3+^ results in the emission of strong persistent infrared light at 696 nm [28].

### Synthetic methods of PLN materials

#### High-temperature solid-state method

The use of the high-temperature solid-state reaction method for the preparation of long-persistent phosphor materials is a relatively traditional approach with widespread application potential. Generally, the solid-state reaction involves the use of solid powders as raw materials [29]. Materials of the required purity are weighed in specific proportions, and a certain amount of flux is added to achieve thorough mixing and grinding. Subsequently, the mixture undergoes calcination under specific conditions (temperature, atmosphere, time, etc.). The specific formulation of the luminescent material according to chemical stoichiometry involved placing the material in a high-temperature resistance furnace, where it is heated under a protective or reducing atmosphere in the range of 900 to 1450°C for 2 to 5 h, resulting in the desired product. The calcination process, choice of flux, and type and ratio of dopant ions all significantly influence the structure and luminescent properties of long-persistent phosphor materials. Due to the increasing optimization of reaction conditions, use of reducing agents, choice of flux, and preparation of raw materials in the high-temperature solid-state method, the production process has matured and is widely applied. For example, long-persistent red phosphor materials in sulfide systems are prepared by thoroughly mixing and calcining alkaline earth metal carbonates, sulfur powder, selected rare earth oxides, and flux. Alternatively, the process involves directly using alkaline earth metal sulfates, rare earth oxides, and flux, followed by uniform mixing and calcination. However, in traditional solid-state annealing reactions, only micron-sized persistent luminescent powders can be synthesized. To convert these bulk crystals into well-dispersed nanoparticles suitable for biological applications, physical treatments such as grinding or laser ablation are needed. However, posttreatment nanoparticles are typically nonuniform in size and prone to aggregation. Therefore, the template method is employed using mesoporous silica as a substrate. Through ion implantation, PLNs are sintered within the pores of silica. Mesoporous silica nanospheres are favored for their controllable synthesis and shape, high surface area, high pore-loading capacity, and excellent biocompatibility; thus, they are widely used in biology, drug delivery, and medical applications for encapsulating various functional drugs or optical contrast agents. The unique optical materials synthesized as PLNs on mesoporous silica substrates continue to emit light after excitation stops. This distinct optical functionality enables luminescent detection without the need for continuous external irradiation, thereby avoiding interference from endogenous fluorescence and scattered light in biological tissues.

#### Sol-gel method

In response to the drawbacks of high-temperature solid-state methods, such as high calcination temperatures and the formation of coarse particles in luminescent materials after ball milling, various alternative methods have been developed. Among these methods, the sol-gel method is a wet chemical approach and has garnered widespread attention in the materials science community. Originating in the eighteenth century, this method has found broad applications. The sol-gel method involves the use of specific precursor materials for hydrolyzing and forming a sol under certain conditions. The sol is then transformed into a network-like gel structure through solvent evaporation and heat treatment. Subsequent appropriate posttreatment processes yield nanostructured materials. The basic process for preparing nanostructured materials using sol-gel technology is as follows: Raw Materials→Dispersible System→Sol→Gel→Nanostructured Material.

The sol-gel technique for preparing luminescent materials primarily utilizes the metal alkoxide method, where metal alkoxides serve as precursors for hydrolysis and polycondensation reactions to form sols and gels. Zhang Dong and colleagues utilized the sol-gel method to prepare ZnAl_2_O_4_:Mn materials, achieving a lower sintering temperature by 100 to 200°C than traditional methods. In recent years, the use of inorganic salt complexes for preparing sol-gels has gained attention, with Pechin’s method being a popular choice. This involves using citric acid and ethylene glycol to undergo esterification, resulting in a sol. This method is quick, simple, and cost-effective compared to metal alkoxide methods [30].

#### Combustion method

The combustion method refers to a process in which materials are synthesized by burning precursors. When the reactants reach the ignition temperature of an exothermic reaction, ignition occurs through a certain method, and subsequent heat release sustains the reaction. The combustion products are the intended materials. The main principle of this method involves preparing reactants as corresponding nitrates, adding urea as a fuel (reducing agent), and heating at a specific temperature for a few minutes. Vigorous oxidation-reduction reactions release a large amount of gas, leading to combustion. Within a few seconds, foamy materials resembling the desired product are obtained and are easily pulverized. This method is highly versatile, and the gases generated during combustion can also serve as a protective atmosphere [31]. The general operation is as follows:

1. The nitrates of SrCO_3_, Al(NO_3_)_3_, Eu_2_O_3_, and Dy_2_O_3_ are mixed in a certain chemical ratio.
2. The appropriate urea and boric acid were added to the mixture.
3. The mixture was dissolved and quickly transferred to a muffle furnace preheated to around 600°C.
4. After the rapid evaporation of water, vigorous oxidation-reduction reactions occur, resulting in combustion and gas overflow.
5. The entire process is short, lasting only a dozen seconds.
6. The product is obtained after combustion, removed, and cooled before grinding.

This method significantly reduces the furnace temperature and is an efficient and energy-saving approach. In addition to the abovementioned methods for the preparation of long-persistent phosphor materials, other techniques, such as hydrothermal synthesis, microwave-assisted synthesis, and chemical precipitation, have also been employed. The use of these novel synthesis technologies has led to breakthroughs in improving the luminescent performance of materials and may yield luminescent materials that traditional preparation techniques cannot achieve, thereby expanding the scope of research and application for long-persistent phosphor materials.

### Luminescence mechanism of PLN materials

In the context of persistent luminescent phosphors, two major concepts are the emission centers (emitters) and traps. Emitters are the centers and, when excited by light, the emitters can radiate; however, traps typically do not possess radiative capabilities. Traps serve to store excited electrons, releasing them gradually into emitters through thermal or other physical stimuli. The radiative wavelength of persistent luminescent phosphors is primarily determined by the emitters, while the persistence intensity and duration are dictated by the trap states, including the trap type and distribution; these are generally associated with lattice defects or co-doped elements.

#### Hole transfer model

**Fig. 2:**
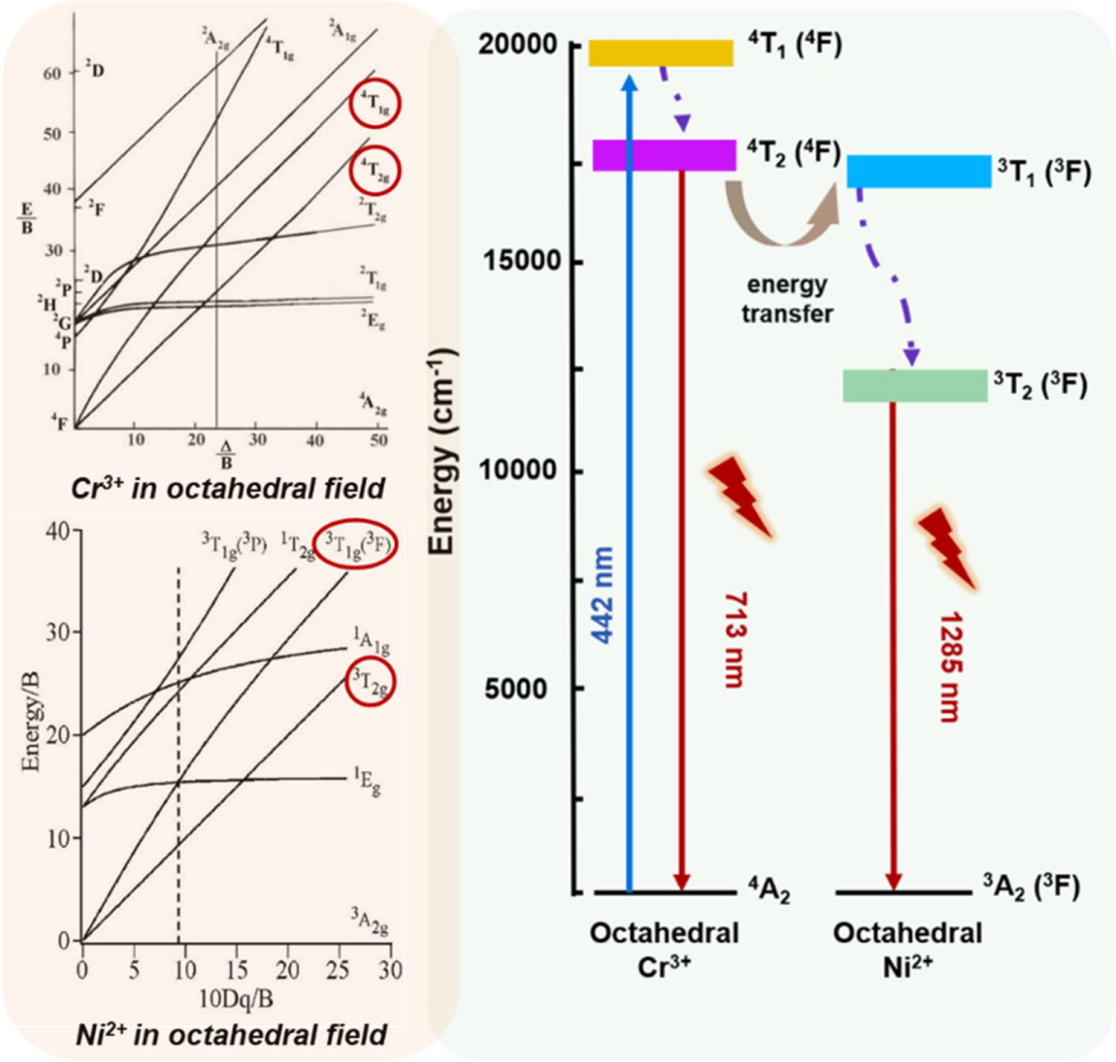
Energy diagram showing energy transfer between the Cr^3+^ and Ni^2+^ sites to achieve the spectrum of PLNs shift from NIR-I to NIR-II. Relative red circles at different energy states of Cr^3+^ spin allowed transitions to Ni^2+^

For these materials, the earliest model was the hole transfer model proposed by Matsuzawa et al*.* for the SrAl_2_O_4_:Eu^2+^,Dy^3+^ system. According to this model, Matsuzawa suggested that in the long afterglow material SrAl_2_O_4_:Eu^2+^,Dy^3+^, Eu served as an electron trapping center, and Dy acted as a hole trapping center [32]. Upon UV excitation, Eu^2+^ captured an electron to become Eu^+^ and the resulting hole was trapped by Dy^3+^ to form Dy^4+^. After excitation ceased, due to thermal motion, the hole escaped, and through a process opposite to the earlier one, it led to the characteristic emission of Eu. Such energy transfer conversion can also be applied to Cr^3+^ and Ni^2+^, as depicted in Fig. 2. This model has been widely cited in the mechanistic explanation of various long afterglow materials co-doped with Cr and Ni, becoming a general interpretation for the mechanism of long afterglow materials co-doped with Cr and Ni.

#### Displacement coordinate model

The displacement coordinate model was initially proposed by Qiu Jianrong and Su Qiang et al*.* Fig. 2 shows (A) schematic diagram of the displacement coordinate model; here, (A) represents the ground-state energy level of Eu^2+^, (B) represents its excited-state energy level, and (C) represents the defect level. C can be impurity ions introduced or defect levels generated in the matrix [33, 34]. Su Qiang and colleagues believed that C can act as an electron-capturing site [35]. Under the action of an external light source, electrons are excited from the ground state to the excited state (1), and some electrons transition back to the low-energy state, emitting light (2). Another portion of electrons is stored in the defect level and C through the relaxation process (3). When electrons in the defect level absorb energy, they are re-excited back to the excited state level, transitioning back to the ground state and emitting light. The duration of the afterglow is related to the number of electrons stored in the defect level and the amount of absorbed energy (heat); more electrons in the defect level result in a longer afterglow time, and more absorbed energy can generate sustained luminescence.

#### Luminous traps extension of the light emission time

**Fig. 3:**
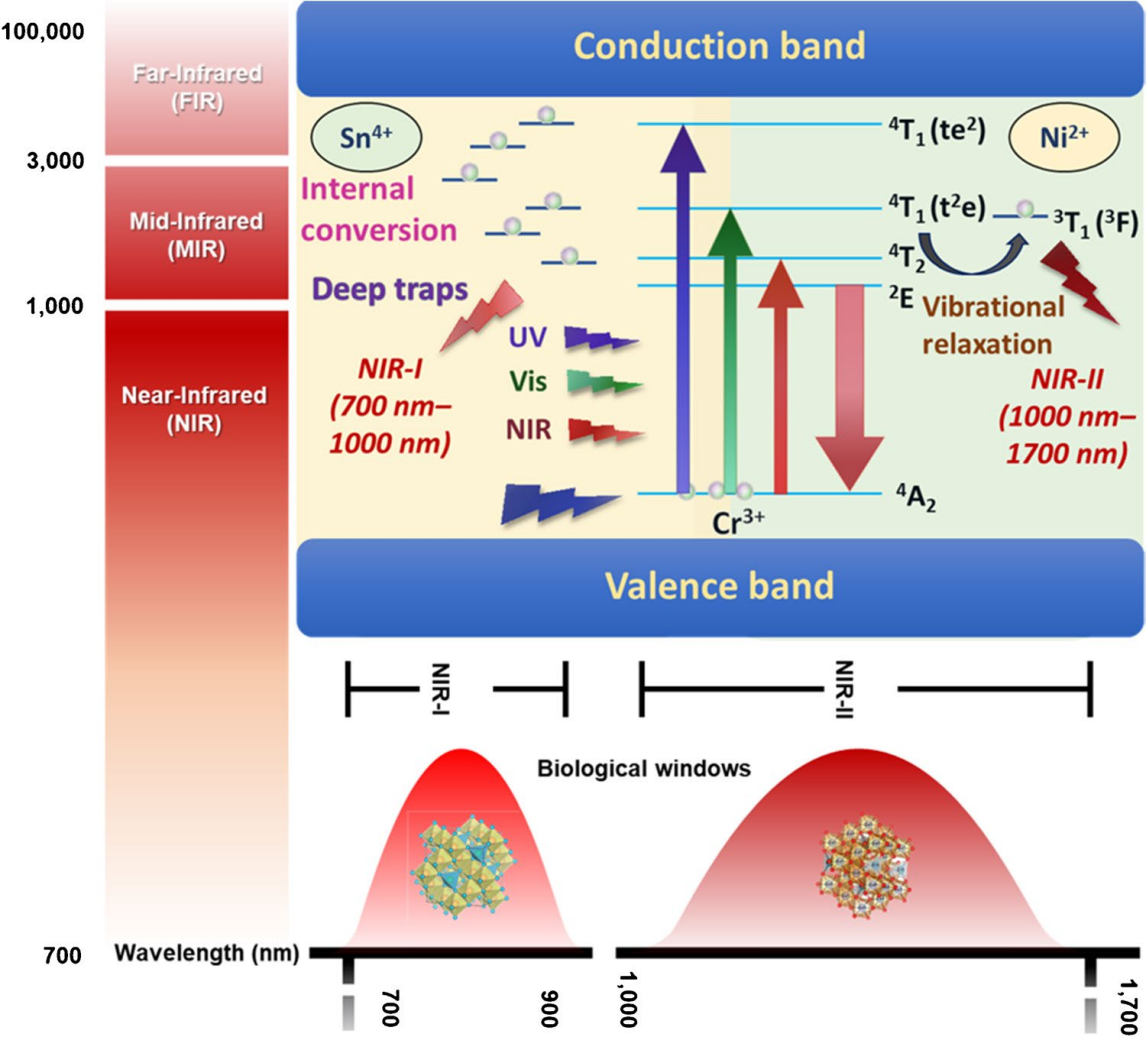
Schematic diagram of the electronic excitation and emission of NIR-I/NIR-II PLNs based on Sn^4+^ doping to make luminous traps and prolong the emission lifetime of NIR persistent luminescent light

In the design of NIR-PLNs, Cr^3+^ is an excellent activator of solid-state luminescent materials that emit NIR light. The emission characteristics, whether narrowband (typically approximately 700 nm due to spin-forbidden ^2^E→^4^A_2_ transitions) or broadband (650–1600 nm due to spin-allowed ^4^T_2_→^4^A_2_ transitions), depend on the crystal field environment of the host lattice. In the development of NIR-PLNs using Cr^3+^ as the luminescent center, the spinel structure of ZnGa_2_O_4_ is frequently used as the host lattice because Cr^3+^ can replace the distorted octahedral positions of Ga^3+^; additionally, the surrounding crystal field strength is suitable to achieve strong NIR light emission. By detecting the 695-nm emission from Cr^3+^, broad excitation bands centered at 271 nm (^4^A2 → ^4^T1(*t*_*2*_^*2*^*e*)), 430 nm (^4^A2 → ^4^T1(*t*_*2*_^*2*^*e*)), and 564 nm (^4^A2 → ^4^T2(*t*_*2*_^*2*^*e*)) in the ultraviolet-visible region were observed, as shown in Fig. 3 [36]. The broad excitation bands at 430 nm and 564 nm correspond to spin-allowed transitions of Cr^3+^, while the 271-nm broad excitation in the ultraviolet region is attributed to the host absorption, indicating energy transfer from the host lattice to Cr^3+^. Due to their ability to be excited by various light sources and emit persistently stable infrared light, as depicted in Fig. 3, these materials have extensive applications in biomedical imaging and therapy. To enhance the luminescence intensity and prolong the exposure time, Sn^4+^ co-doping is utilized. This co-doping not only increases antisite defects, extending the luminescence, but also induces Ga^3+^ octahedral site distortions, leading to self-recombination of excited states between neighboring defects. This mechanism generates positive defects and results in the formation of effective deep traps.

## NIR-I/II persistent luminescent nanoparticles for biosensing and bioimaging

PLNs are widely used in optical sensing and detection due to their special luminescence phenomenon, ultra-long afterglow lifetime, the ability to avoid *in situ* excitation, and the ability to control the spectral emission region within the “bio-optical transparent window.” In recent years, the synthesis and application of PLN probes have attracted great attention in the fields of spectroscopy, phononics, photochemistry, and materials science. This section conducts an in-depth discussion on the synthesis methods of PLN probes, functionalization of particle surfaces, and their application as targeted probes for sensing detection and *in vivo* imaging. According to the characteristics of infrared light, PLNs are divided into luminescent regions doped with Cr^3+^, Mn^2+^ (NIR-I), and Ni^2+^ (NIR-II). Functionalized NIR-PLNs provide a promising technology platform for long-term real-time monitoring of physiological processes *in vivo* and diagnosis of diseases.

Another important applicability of NIR-PLNs is fluorescence imaging, characterized by its noninvasiveness, high sensitivity, and superior spatiotemporal resolution, which has found widespread applications in fields such as life sciences and clinical medicine [37]. In comparison to the visible light window (400–650 nm), biological tissues exhibit reduced absorption and scattering of both excitation and emission light in the NIR-I window (650–900 nm) and NIR-II window (1000–1700 nm). Consequently, the optical signals within the NIR-II window significantly enhance the penetration depth, resolution, and signal-to-noise ratio for *in vivo* imaging [38]. Moreover, recent clinical studies indicate that fluorescence imaging in the NIR-I and NIR-II windows can guide precise tumor resection surgeries, presenting broad prospects for clinical applications [39]. However, traditional fluorescence imaging relies on real-time excitation of fluorescent probes within biological organisms using external light sources and inevitably results in background fluorescence from biological tissues that impacts imaging resolution and the signal-to-noise ratio. Moreover, the irradiation of external light sources can potentially lead to overheating, posing a risk of damage to biological tissues. Therefore, enhancing the resolution and signal-to-noise ratio of *in vivo* optical images and obtaining accurate imaging information remain challenging for researchers [40]. PLNs in the NIR-I and NIR-II regions exhibit deep tissue penetration, and they can be re-excited by NIR light to emit light for an extended period, overcoming limitations on the emission duration [41]. To fully harness PLNs for biological applications, strategies for bridging PLNs with biological systems need to be designed.

**Fig. 4:**
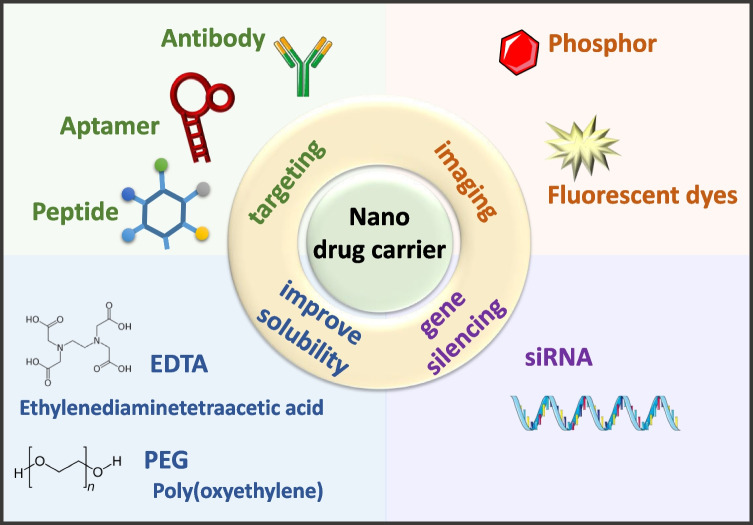
Applications of PLNs in biological imaging, biosensors, and therapeutics

**Fig. 5:**
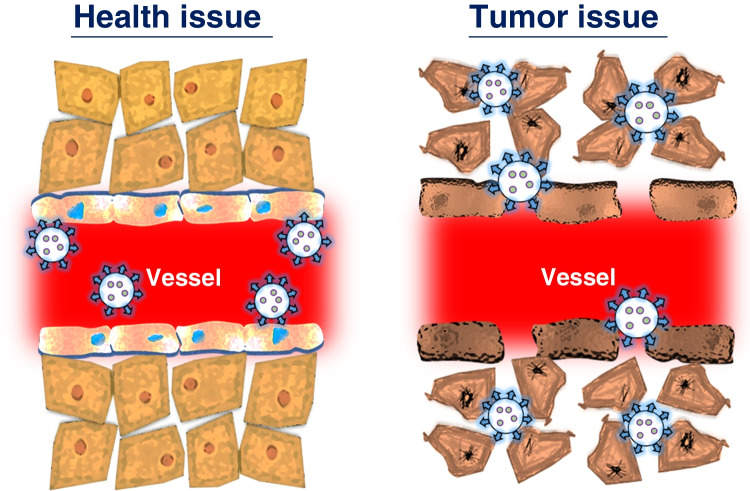
EPR effect leading to the accumulation of nanomaterials in tumor tissues

Through surface modification of PLNs and energy transfer between modifiers and targets, applications in biosensors, targeted imaging, multimodal imaging, theranostics, and other fields can be achieved, as illustrated in Fig. 4. Since current research has limited the size of afterglow materials to the level of nanometerization, these materials have also benefited from the advantages of nanotechnologies, such as high permeability and long residence times. The enhanced permeability and retention (EPR) effect is one of the principles underlying the delivery of drugs using nanocarriers. EPR allows drug carriers to passively accumulate in tumor tissues and can be combined with tumor treatment methods such as photothermal therapy, photodynamic therapy, and chemotherapy to achieve better therapeutic effects. EPR refers to the phenomenon in which macromolecules of specific sizes (such as nanoparticles, liposomes, or macromolecular drugs) easily penetrate tumor tissues and exhibit a prolonged retention effect. This phenomenon arises from the rapid proliferation of tumor cells, which induce the growth of tumor blood vessels by secreting vascular endothelial growth factor (VEGF) to obtain nutrients and oxygen. When the tumor reaches a size of 150–200 μm, the vascular structure of the tumor differs from that of normal blood vessels. Endothelial cells rapidly proliferate, leading to a decrease in the number of pericytes and a lack of a smooth muscle layer in the vessel wall. Additionally, compared to normal blood vessels, which have junctions of only 5 to 10 nm, angiotensin receptor function becomes impaired, causing the tumor blood vessel structure to have larger pores with a diameter range of several hundred nanometers. These large pores result in increased vascular permeability and hydraulic conductivity in tumor tissues, enabling large macromolecules to pass through the vessel wall and passively accumulate in the tumor tissue. Furthermore, tumor tissues lack lymphatic vessels, hindering lymphatic fluid drainage. As a result, large macromolecules are not drained back into the bloodstream by lymphatic fluid and can persist in the tumor tissue for an extended period, as illustrated in Fig. 5.

### Biomedical imaging applications of PLN materials

Due to its advantages of high sensitivity and simplicity, fluorescence detection is widely used in basic research and clinical applications. However, traditional fluorescence probes suffer from poor signal-to-noise ratios due to interference from endogenous fluorescence and light scattering in biological matrices under constant external excitation [42]. NIR-PLNs can eliminate interference from endogenous fluorescence; additionally, when pre-excited before detection, PLNs can mitigate the impact of light scattering [43]. This approach addresses the issue of high background signals in the traditional fluorescence biosensors. Considering the chemical inertness and excellent photostability of NIR-PLNs, the design of a fluorescence resonance energy transfer (FRET) system using these nanoparticles is the most suitable strategy for manufacturing nanoprobes [33, 44–46]. Multimodal imaging, which combines different imaging modes, enables simultaneous high-sensitivity and high-resolution diagnosis. By combining the unique advantages of NIR-PLNs with those of multimodal imaging, high-performance NIR-PLN imaging nanoprobes can be designed (Table 1). Used in magnetic resonance imaging, NIR-PLNs provide physiological and anatomical information with high spatial resolution [47]. For instance, NIR-PLNs decorated with clinical magnetic resonance imaging contrast agents (Gd-DTPA) maintain excellent NIR persistent luminescence while enhancing the surface relaxation of Gd-DTPA, resulting in high sensitivity and good spatial resolution [48].

**Fig. 6:**
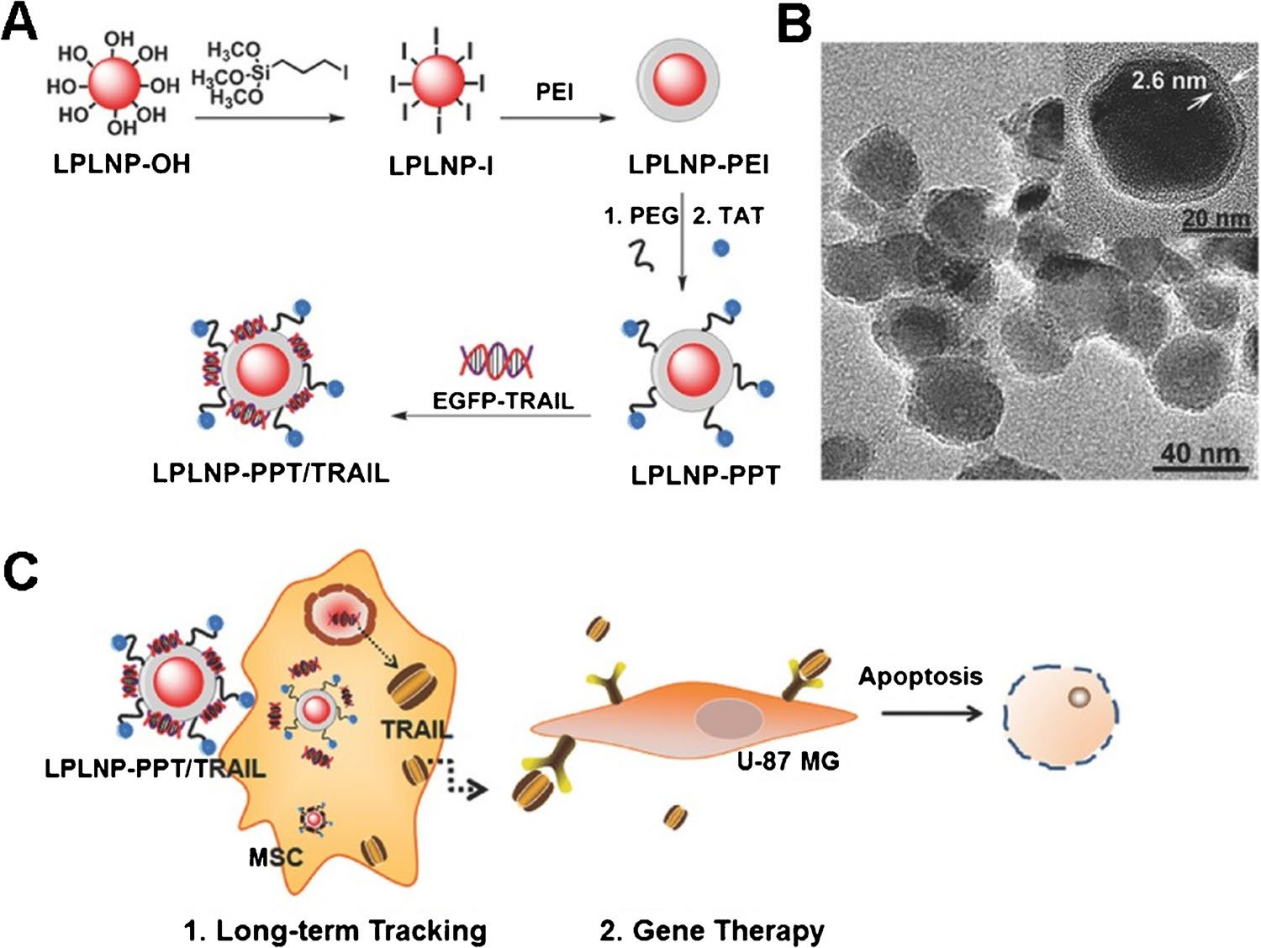
Nanocomposite material of mesenchymal stem cells and NIR-PLNs for the homing effect and gene therapy of glioblastoma. **A** The modifications of PLNs to enhance the solubility of the particles. **B** The TEM images demonstrate the surface modifications of PLNs. **C** The schematic figure makes the tracking of cells with persistent luminescent signals. Reproduced with permission [66]. Copyright 2017, Wiley Online Library

The persistent luminescent property of NIR-PLNs is also widely applied in tumor therapy and serves as an internal light source for photodynamic therapy [64]. In photodynamic therapy, a photosensitizer generates singlet oxygen (^1^O_2_) or reactive oxygen species upon light exposure to eradicate tumors. This therapy has been clinically approved for treating various tumors [50]. However, the limited penetration depth of external light sources in biological tissues restricts photodynamic therapy for deep tissue tumor treatment. Additionally, continuous light exposure poses a risk of tissue overheating damage, hindering the widespread application of photodynamic therapy [65]. NIR-PLNs with persistent and repeatable luminescence capabilities can serve as internal light sources for photodynamic therapy without the need for continuous external irradiation, thus preventing tissue damage [54]. Furthermore, the persistent luminescent property of NIR-PLNs is utilized in photothermal therapy by generating heat energy from their luminescence. This approach is employed to eliminate tumor cells with the use of biocompatible photothermal therapeutic agents (such as copper sulfide nanoparticles). Connecting NIR-PLNs and copper sulfide nanoparticles via a matrix metalloproteinase (MMP)-sensitive peptide substrate creates an activated MMP therapeutic system. This system has high selectivity and minimal invasiveness. Due to the unique advantage of NIR-PLNs, coupled with their ability for multimodal imaging, they are versatile tools for various applications, such as drug localization tracking and tumor cell tracking in the biomedical field. In comparison to magnetic resonance imaging or computed tomography imaging, optical imaging is highly sensitive, nonradioactive, minimally invasive, easy to perform, and cost-effective; thus, it is a potent tool for biological imaging. However, when using common nanofluorescent particles or organic dyes as markers, significant background autofluorescence interference occurs, and these materials are prone to *in situ* excitation. To address these challenges, NIR-PLNs have been proposed for glioblastoma treatment. Wu et al*.* designed a nanocomposite material combining mesenchymal stem cells (MSCs) and near-infrared persistent luminescent nanoparticles (LPLNPs) for the homing effect and gene therapy of glioblastoma, as illustrated in Fig. 6 [66]. This nanocomposite material effectively induced the transfection of MSCs to express therapeutic TRAIL ligands without affecting their proliferation, differentiation, and tumor-homing ability. It could also efficiently track long-term changes in MSCs in glioblastoma without the need for continuous external light excitation.

**Fig. 7:**
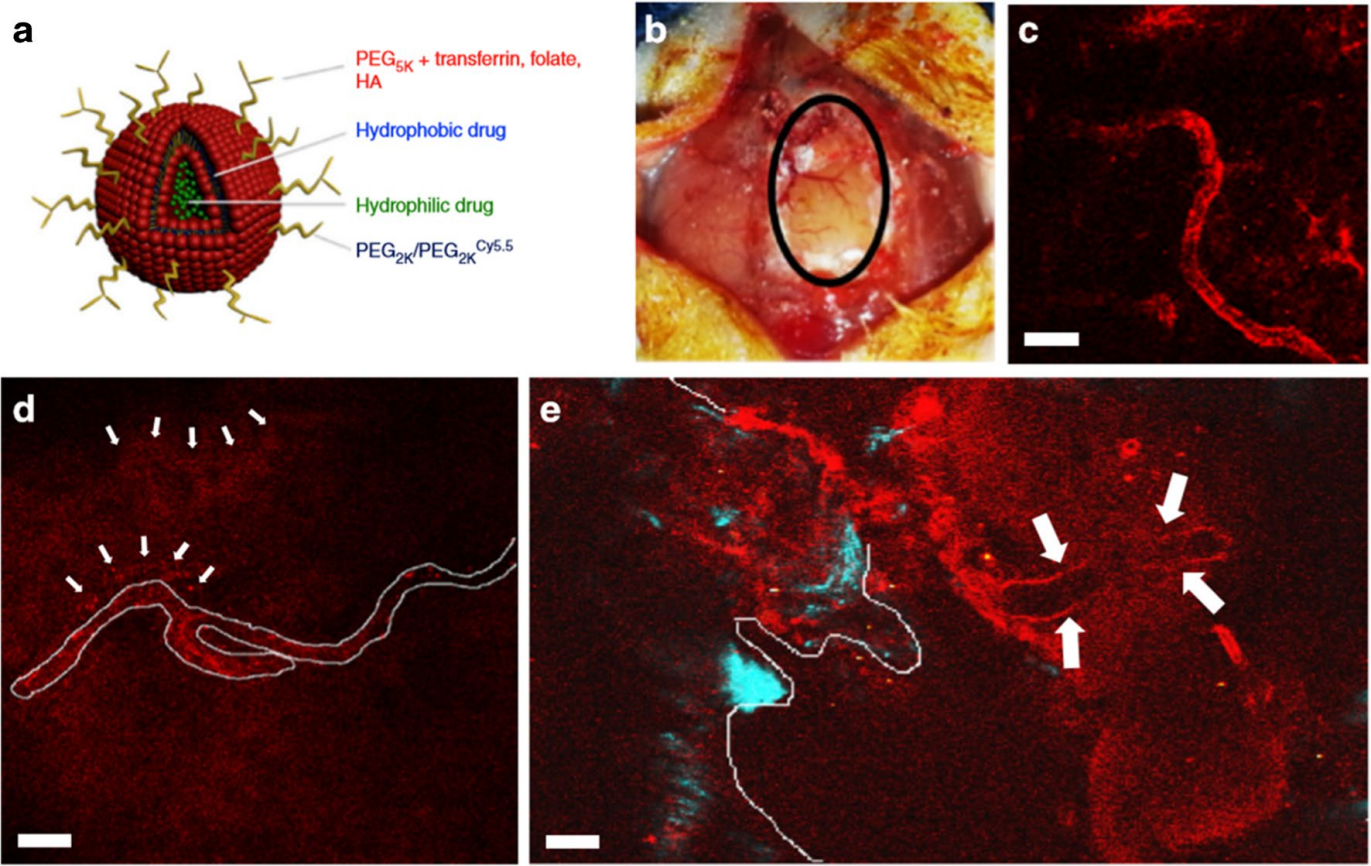
PLNs surface-modified with transferrin providing dual-drug combination therapy for glioblastoma. **a** Modification of PLNs through targeting molecules. **b** The brain tissue is surgically dissected and the intracerebral vascular structure is observed using optical imaging. **c**–**e** NIR images through PLNs show intracranial blood vessels beneath glioblastoma. Reproduced with permission [67]. Copyright 2018, Springer Nature Limited

Treatment for glioblastoma is limited by the blood-brain barrier and rapid resistance to monotherapy. To overcome these issues, Lam et al*.* developed nanoparticles surface-modified with transferrin (Tf-PLNs) to provide dual-drug combination therapy, as shown in Fig. 7 [67]. Tf-PLNs effectively crossed the blood-brain barrier in mice, and in two intracranial *in situ* models of glioblastoma, Tf-PLNs were directly bound to the tumors. The chemical drug temozolomide and the bromodomain inhibitor JQ1 were simultaneously administered to treat glioblastoma in mice, which resulted in DNA damage and apoptosis. Compared to an equivalent dose of free drugs, the tumor size was reduced by 1.5 to 2 times [68]. Treatment of immunocompetent mice with drug-loaded Tf-NPs also had a protective effect on systemic drug toxicity, demonstrating the potential of the nanocomposite material for novel combination therapy for glioblastoma and other central nervous system tumors. Moreover, the NIR-II region refers to the wavelength range of 1000–1700 nm. Within this wavelength range, there is lower autofluorescence, scattering, and absorption in biological tissues. Additionally, light in this wavelength range has better penetration, especially considering the significant differences in light scattering in the brain. Moreover, the NIR-II region allows for a more diverse range of fluorescent materials to be used. Besides traditional small molecule fluorophores and quantum dots (QDs), NIR-PLNs materials also exhibit fluorescence characteristics in the infrared II region. They hold the potential to be developed into novel biosensors, biomedical imaging tools, and applications in photodynamic therapy. Satpathy et al*.* developed a novel NIR-II PLNs to obtain the highest emission intensity in the NIR-II region at 1285 nm via energy transfer from Cr^3+^ to Ni^2+^ [60]. These NIR-PLNs establish emission in the NIR-II and NIR-I regions to obtain images *in vivo* with an increased penetration depth to 5 mm and such NIR-II optical imaging can be achieved on mouse system. Based on the above research and studies, it can be expected that the novel NIR-PLNs system will open the door for optical bioimaging research on other NIR-I and NIR-II nanophosphors.

### Biosensing applications of PLN materials

Fluorescent chemical sensors, utilizing fluorescence signals as output information, are capable of detecting specific recognition of target molecules. They have found extensive applications in various fields, including food, biology, chemistry, the environment, and medicine. In comparison to traditional fluorescent dyes, fluorescent nanomaterials possess numerous advantages, such as high fluorescence intensity, good optical stability, and easy surface modification; these have led to significant advancements in the development of fluorescent chemical sensors [58]. Conventional fluorescent nanomaterials include noble metal nanoclusters, upconversion nanomaterials, quantum dots, and carbon dots [34, 69, 70]. These materials share a common drawback: the need for constant excitation, preventing real-time detection of target substances. This limitation significantly affects their application in real-time detection. Conversely, PLNs do not require *in situ* excitation. Due to this characteristic, PLNs can avoid the autofluorescence generated by biological tissues, reduce background noise, enhance the signal-to-noise ratio, and improve sensitivity (Table 2). After surface functionalization, PLNs can be applied in various fields, including biosensing, cellular and *in vivo* imaging, drug delivery, and therapy.

**Table 2:** Biosensing applications of PLN materials

Optical windows	PLNs material	Combined materials	Conjugation	Target	Limit of detection	Ref.
Near-infrared-I (650–950 nm) (from 2018 to 2023)	ZnGa_2_O_4_:Cr^3+^-amorphous calcium phosphate (^+^CZGO–ACP)	n/a	Polyacrylic acid (PAA)	Ca^2+^ and PO_4_^3−^	n/a	[17]
	Zn_2_Ga_3.98-4*x*/3_Ge_*x*_O_8_:Cr_0.02_	n/a	n/a	Temperature	1.0% K^−1^	[58]
	ZGO–EDTA	n/a	EDTA	Metals (mainly Ga and Zn	1713±38 441±1 (mg L^−1^)	[71]
	ZnGeO:Mo	Polydopamine nanoparticles (PDANSs)	Aptamers	Prostate-specific antigen (PSA)/carcinoembryonic antigen (CEA)	8.9 fg mL^−1^/72 fg mL^−^	[72]
	ZnGa_2_O_4_:Cr^3+^	Hierarchical porous zeolite imidazole framework-8 (HZIF-8)	n/a	Dopamine	0.0025–75 μM	[73]
	ZCGO	Mesoporous silica	Manganese dioxide (MnO_2_)	Glutathione	0.62 μM	[74]

^*^n/a means “Not applicable”

**Fig. 8:**
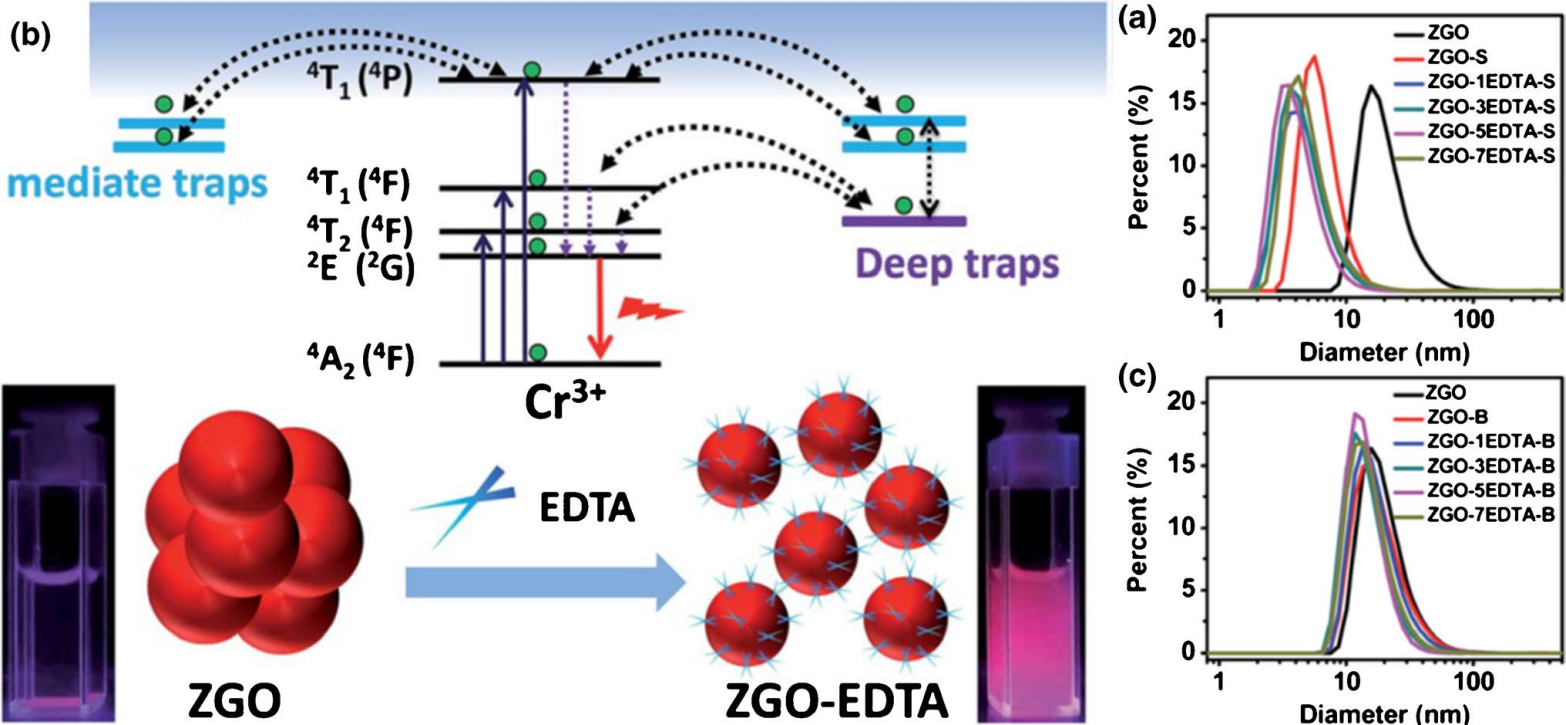
PLNs have aroused considerable attention in pre-excitation optosensing, via a simple post-treatment of ethylenediaminetetraacetate (EDTA) etching. **a** The size distribution of ZnGa_2_O_4_:Cr^3+^. **b** The schematic figure demonstrates the EDTA sensing by PLNs. **c** The suspension stability of ZnGa_2_O_4_:Cr^3+^-nEDTA-B. Reproduced with permission [71]. Copyright 2019, Royal Society of Chemistry

In recent years, the field of biosensing has witnessed remarkable advancements, driven by the integration of innovative nanomaterials into sensing platforms. Among these materials, PLNs have emerged as a promising candidate, offering unique advantages for sensitive and prolonged detection of biological analytes. Unlike traditional fluorescent probes that quickly lose their emission after excitation, PLNs exhibit a remarkable capability to emit light over extended periods without the need for continuous excitation. This distinctive feature makes them particularly well-suited for biosensing applications where prolonged signal stability and minimal interference are crucial. This characteristic allows them to avoid the autofluorescence generated by biological tissues, reducing background noise, enhancing the signal-to-noise ratio, and improving sensitivity (Table 2). After surface functionalization, PLNs can be applied in various fields as suitable biological sensors. Wang et al*.* used the quantum trap provided by EDTA to detect the concentration and content of EDTA in organisms (Fig. 8) [71]. In addition to the suitable optical sensitivity time of PLNs produced by transition metals, the tunability of lanthanide metals has also become an advantage for biomolecule sensing. The foundation of PLNs lies in their composition, typically comprising host matrices doped with rare earth ions. This doping imparts exceptional photostability to the nanoparticles, allowing them to retain luminescence long after the excitation source is removed. The prolonged emission of PLNs offers a distinct temporal advantage over conventional fluorophores, enabling enhanced signal-to-noise ratios and prolonged observation windows for biosensing applications. In multicolor lanthanide-doped CaS and SrS NIR PLNs, Qingyun Cai’s research team demonstrated that their lanthanide-doped PLNs can sense organic ligands such as 1-dodecanethiol (DT) and 11-mercaptoundecanoic acid (MUA). As we delve deeper into the diverse avenues of PLN-based biosensing, it becomes evident that these luminescent nanomaterials hold immense promise for shaping the future landscape of sensitive and enduring detection methodologies.

PLNs have emerged as promising candidates for biosensing applications due to their unique optical properties and potential for long-lasting luminescence. However, the biosensing capabilities that PLNs alone can provide are still limited. Thus, PLN nanocomposites, a class of materials composed of nanoscale components dispersed within a matrix, offer a hybrid of advantages across various fields due to combining different properties of different nanoparticles and versatile applications. By harnessing the persistent luminescence emitted by these nanocomposites, researchers can develop highly sensitive and selective biosensors capable of detecting even trace amounts of target analytes in complex biological samples. Shi et al*.* used Zn_2_GeO_4_ host PLN material that combines with polydopamine nanoparticles (PDANSs) for the dual detection of prostate-specific antigens (PSA) and carcinoembryonic antigens (CEA) [72]. These dual persistent luminescence signals at 534 and 696 nm both can be quenched by PDANSs and then recovered in the presence of PSA and CEA. These developed “turn-on” luminescence sensors achieved accurate and sensitive autoluminescence-free screening of PSA and CEA in the real serum matrix.

## Applications of nanocomposite build with NIR-I/II persistent luminescent nanoparticles

PLNs are composed of a host material and dopant elements. The host material is similar to semiconductor materials, providing a valence band and a conduction band. However, inherent structural defects, such as vacancies, naturally exist in the lattice of host materials. Vacancies imply the disappearance or displacement of atoms that should be present in the lattice, causing discontinuous energy levels in the valence and conduction bands. These energy levels serve as electron traps, and upon light excitation, electrons move from the conduction band to these traps, gradually filling them. After ceasing light excitation, a significant number of electrons return from the conduction band to the emission centers, emitting fluorescence, while a small fraction of the electrons undergo electron tunneling, return to the emission centers, slowly combine with holes, and generate long-lasting luminescence. Dopant elements serve two purposes: increasing the number of electron traps, such as Pr^3+^, Yb^3+^, and Er^3+^, and acting as emission centers, such as Cr^3+^. The emission centers influence the absorption and emission wavelengths as well as the luminescence intensity of the nanoparticles. Moreover, PLNs exhibit the characteristics of prolonged luminescence and continuous tracking, avoiding autofluorescence and reducing noise interference. Due to their superior tissue penetration of infrared light, PLNs with infrared emission are commonly applied in biomedical applications, such as cellular tracking and phototherapy.

**Fig. 9:**
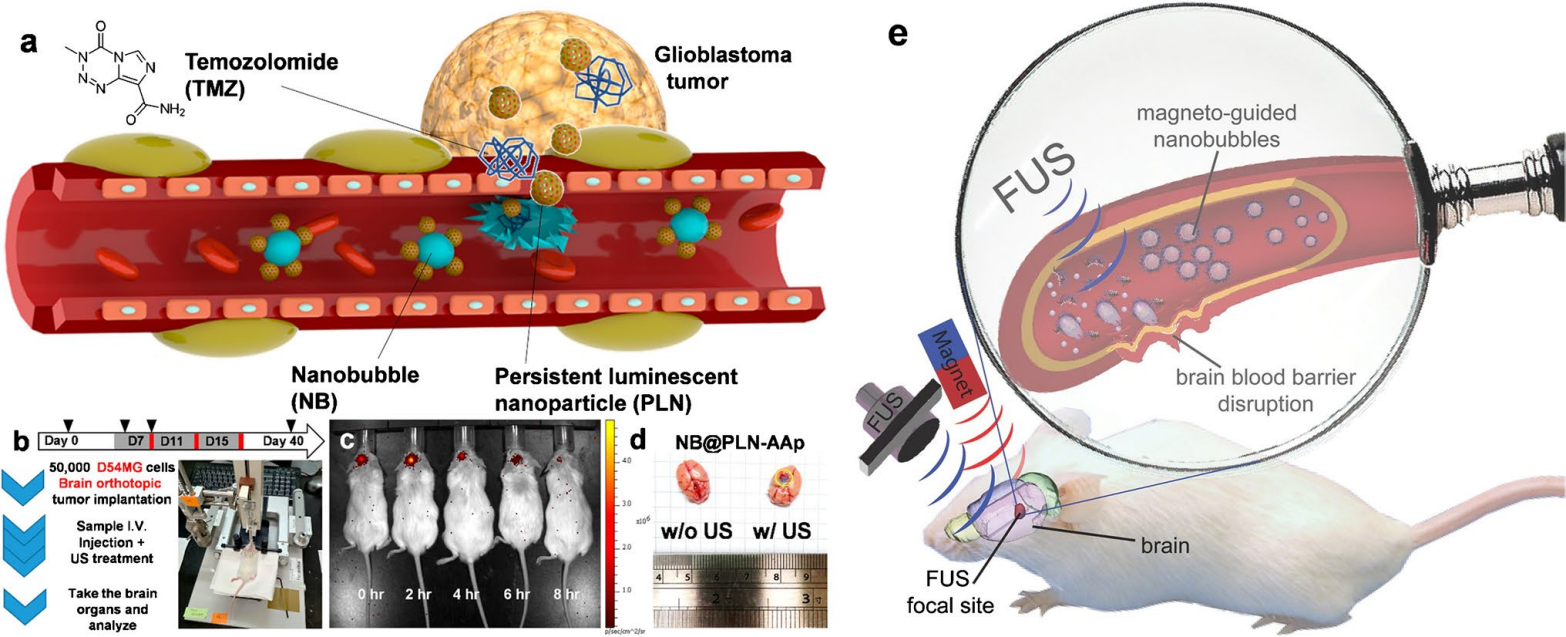
Ultrasound-induced glioblastoma treatment for optimized PLNs targeted delivery. **a** Design of the PLNs nanosystem. **b** The process of building glioblastoma mice model. **c** PLNs NIR tracking imaging. **d** Using the cavitation method to make the PLNs penetrate the blood-brain barrier. **e** Magnetic guidance of nanobubbles increases their concentration in the brain region to open the blood-brain barrier. Reproduced with permission [76, 82]. Copyright 2015 and 2021, American Chemical Society and Wiley Online Library

The scope of biotechnology research on nanocomposite materials encompasses crucial areas such as nanomaterials, nanodrug carriers for drugs and transgenes, nanobiocompatible artificial organs, nanobiosensors, and imaging technologies for analyzing the structure and function of proteins and DNA (Table 3). The primary goal is to achieve early disease diagnosis and enhance therapeutic efficacy. In the realm of nanocomposite materials, research on nanodrug carriers has advanced to the clinical use stage, particularly in the experimental phase of nanobiotechnology applications for malignant tumor diagnosis and treatment. Because of the unique optical characteristics of PLNs, they are very suitable for use in combination with other nanomaterials to form nanocomposites that emit light for a long time. Recent research has shown that bubble-like nanomaterials can be combined with PLNs to form composite materials for long-term biological imaging monitoring (Fig. 9). Nanobubbles have been employed as contrast agents for ultrasound imaging and therapy. However, compared with microbubbles, nanobubbles exhibit relatively lower stability. After being triggered by focused ultrasound, nanobubbles can cause damage to the endothelium or parenchyma due to their intense cavitation effect, leading to massive red blood cell extravasation [82]. Therefore, Cheng et al*.* exploited the advantages of PLNs to enhance the tracking efficiency and overcome the challenges encountered in the application of nanobubbles for diagnosis and treatment [76]. According to previous research, PLNs have low toxicity, can emit light for a long time, and avoid autofluorescence effects, enabling them to be powerful imaging materials that can be used to track the brain states of mice affected by nanobubbles for a long time.

**Table 3:** Applications of nanocomposite build with NIR-I/II PLNs

Optical windows	PLNs material	Combined materials	Conjugation	Functions	Ref.
Near-infrared-I (650–950 nm) (from 2018 to 2023)	Zn_1.25_Ga_1.5_Ge_0.25_O_4_: 0.5%Cr^3+^,2.5%Yb^3+^,0.25%Er^3+^	Gold nanoparticles	AS1411 aptamer	Theragnostic nanoprobe	[75]
	ZnGa_2_O_4_:Cr^3+^,Sn^4+^	Nanobubbles	AS1411 aptamer	Theragnostic nanoprobe	[76]
	Zn_1+x_Ga_2-2x_Ge_x_O_4_:Cr_0.0075_ (x, 0.1–0.5)	Polypyrrole	Polyethylene glycol (PEG)	Dual-modal imaging	[77]
	SrAl_12_O_19_:Fe^3+^	3-Aminopropyl triethoxysilane	n/a	NIR-I Bioimaging	[78]
	CaWO_4_:Eu^3+^,Sm^3+^	Diethyleneglycol	n/a	NIR-I bioimaging	[79]
	ZnGa_2_O_4_:Cr^3+^	Mesoporous silica coating gold nanorods (cGNR@MSNs)	ANTI-CD40-divinylpyrimidine derivatives	Multimodality imaging	[80]
	Zn_2_Ga_2.98_Ge_0.75_O_8_:Cr^3+^_0.02_,Bi^3+^_*x*_	Mesoporous silica	Zinc phthalocyanine	NIR-I bioimaging	[81]

^*^n/a means “Not applicable”

These nanocomposite materials can be used for multifunctional surface modifications and can even be loaded with other drugs to provide simultaneous imaging and treatment effects. Current research is very limited on the development of PLN nanocomposites in the second region of near-infrared light. Even though composite materials have been used to improve the properties of PLN, they have rarely been used in the biomedical field. Therefore, it is anticipated that in future research, these composite nanomaterials can be applied to the treatment and sensing of other diseases and can be used to track disease development. Besides, there are still many aspects that can be optimized in the material itself; these include simplifying the synthesis route and improving the convenience of application of this composite material. These are all research topics that can be explored in the future.

## Conclusions and perspectives

The significant enhancement of therapeutic and tracking efficacy is achieved through the use of composite nanoparticles, leveraging the advantages of different nanoparticles to overcome various obstacles in medical treatment. In terms of application, composite nanoparticles exhibit multiple functionalities and ease of use, whether for drug transport or modification with targeting molecules, with persistent luminescent nanoparticles (PLNs) being particularly suitable materials. PLNs possess low toxicity, long-lasting luminescence, and the ability to avoid autofluorescence; thus, they are powerful imaging agents. The objective is to apply these composite nanomaterials for the treatment of other diseases, aiming to continually optimize the tracking of disease progression. In the future, the outlook for PLNs in biosensing and bioimaging appears promising. Here are some perspectives: (1) Enhanced Sensitivity: Continued research efforts can focus on improving the sensitivity of PLNs for biosensing applications. By refining the synthesis methods and surface functionalization techniques, PLNs could achieve higher sensitivity levels, enabling the detection of biomolecules at even lower concentrations. (2) Multimodal Imaging: PLNs offer the advantage of persistent luminescence, which can enable long-term imaging without the need for continuous excitation. Integrating PLNs with other imaging modalities such as magnetic resonance imaging (MRI) or computed tomography (CT) could provide complementary information and enhance the accuracy of bioimaging. (3) Targeted Delivery: Functionalizing PLNs with targeting ligands or antibodies can facilitate specific binding to disease biomarkers or cellular targets. This targeted delivery approach could improve the precision of drug delivery and enhance the effectiveness of therapeutic interventions. (4) Clinical Translation: Translating PLN-based biosensing and bioimaging technologies from the laboratory to clinical settings will require rigorous evaluation of their safety, efficacy, and regulatory approval. Collaborations between researchers, clinicians, and industry partners will be crucial for overcoming the challenges and accelerating the clinical translation process.

Overall, PLNs hold great potential for revolutionizing biosensing and bioimaging applications, offering new opportunities for disease diagnosis, treatment monitoring, and personalized medicine in the future. Further optimization of the PLN materials is possible, such as simplifying the synthesis pathway and improving the convenience of application for this composite material. These aspects represent ongoing challenges for future development.
